# Supplementary material for: Display of the human mucinome with defined O-glycans by gene engineered cells
Source: Nat Commun. 2021 Jul 1;12:4070. doi: 10.1038/s41467-021-24366-4 (PMC8249670; doi:10.1038/s41467-021-24366-4)
Supplement: Supplementary file 1 — Supplementary Information [file 41467_2021_24366_MOESM1_ESM.pdf]

Supplementary Information

Display of the Human Mucinome with Defined O-Glycans by Gene Engineered Cells

Rebecca Nason[1], Christian Büll[1], Andriana Konstantinidi[1], Lingbo Sun[1], Zilu Ye[1], Adnan Halim[1], Wenjuan Du[2], Daniel M. Sørensen[1], Fabien Durbesson[3] Sanae Furukawa[1], Ulla Mandel[1], Hiren J. Joshi[1], Leo Alexander Dworkin[1], Lars Hansen[1], Leonor David[4,5], Tina M. Iverson[6], Barbara A. Bensing[7], Paul M. Sullam[7], Ajit Varki[8], Erik de Vries[2], Cornelis A.M. de Haan[2], Renaud Vincentelli[3], Bernard Henrissat[1,3,9], Sergey Y. Vakhrushev[1], Henrik Clausen[1*], Yoshiki Narimatsu[1,10*]

1 Copenhagen Center for Glycomics, Departments of Cellular and Molecular Medicine and School of Dentistry, Faculty of Health Sciences, University of Copenhagen, Blegdamsvej 3, Copenhagen, Denmark
2 Section Virology, Division of Infectious Diseases and Immunology, Department Biomolecular Health Sciences, Faculty of Veterinary Medicine, Utrecht University, Yalelaan 1, 3584 CL Utrecht, the Netherlands
3 Architecture et Fonction des Macromolécules Biologiques, CNRS, Aix-Marseille Université, Marseille, France
4 Institute of Molecular Pathology and Immunology of the University of Porto/I35, 4200-465 Porto, Portugal
5 Medical Faculty of the University of Porto, Rua Dr. Roberto Frias, 4200-319, Porto, Portugal
6 Departments of Pharmacology and Biochemistry, Vanderbilt University, Nashville, TN 37232
7 Department of Medicine, The San Francisco Veterans Affairs Medical Center, and the University of California, San Francisco, CA 94121, USA
8 The Glycobiology Research and Training Center, and the Department of Cellular and Molecular Medicine, University of California, San Diego, CA 92093, USA
9 Department of Biological Sciences, King Abdulaziz University, Jeddah, Saudi Arabia
10 GlycoDisplay ApS, Copenhagen, Denmark

Supplementary Figure 1. Design of the human mucin TR reporters. **a** Schematic presentation of the imperfect TR amino acid sequences selected**. b** Parallel plot of key amino acid residues in the human mucin TR reporter designs.

Supplementary Figure 2. Validation of mucin TR and O-glycoform expression.

Supplementary Figure 3. Production of secreted mucin reporter proteins.

Supplementary Figure 4. Immunochemical ELISA analysis of glycoengineered MUC1 and MUC7 TR reporters.

Supplementary Figure 5. HPLC isolation of MUC1 TR O-glycodomains for intact mass analysis.

Supplementary Figure 6. MS analysis of MUC1 TR expressed in GALNT4 KO engineered HEK293$^{KO\ COSMC}$ cells.

Supplementary Figure 7. MALDI-TOF profiling of released MUC1 TR O-glycans.

Supplementary Figure 8. Mucin display reveals binding specificities of *Streptococcus* adhesins.

Supplementary Figure 9. Analysis of StcE on secreted and membrane-bound mucin TR reporters.

Supplementary Figure 10. The StcE X409 domain binds mucins *in situ*.

Supplementary Table 1. Amino acid sequences of mucin TR reporter constructs used, related to Figure 1 and supplementary Figure 1.

Supplementary Table 2. Summary of engineered HEK293 isogenic cell library generated to date, related to Figure 1.

Supplementary Table 3. List of CRISPR gRNA design and PCR primers used in this study.

Supplementary Table 4. A peak list of intact mass spectra.

Supplementary Table 5. A peak list of intact mass spectra of experimentally determined (MW cal) masses related to Figure 3.

Supplementary Table 6. A peak list of intact mass spectra experimentally determined (MW ex) and theoretically calculated (MW cal) masses related to Supplementary Figure 7a.

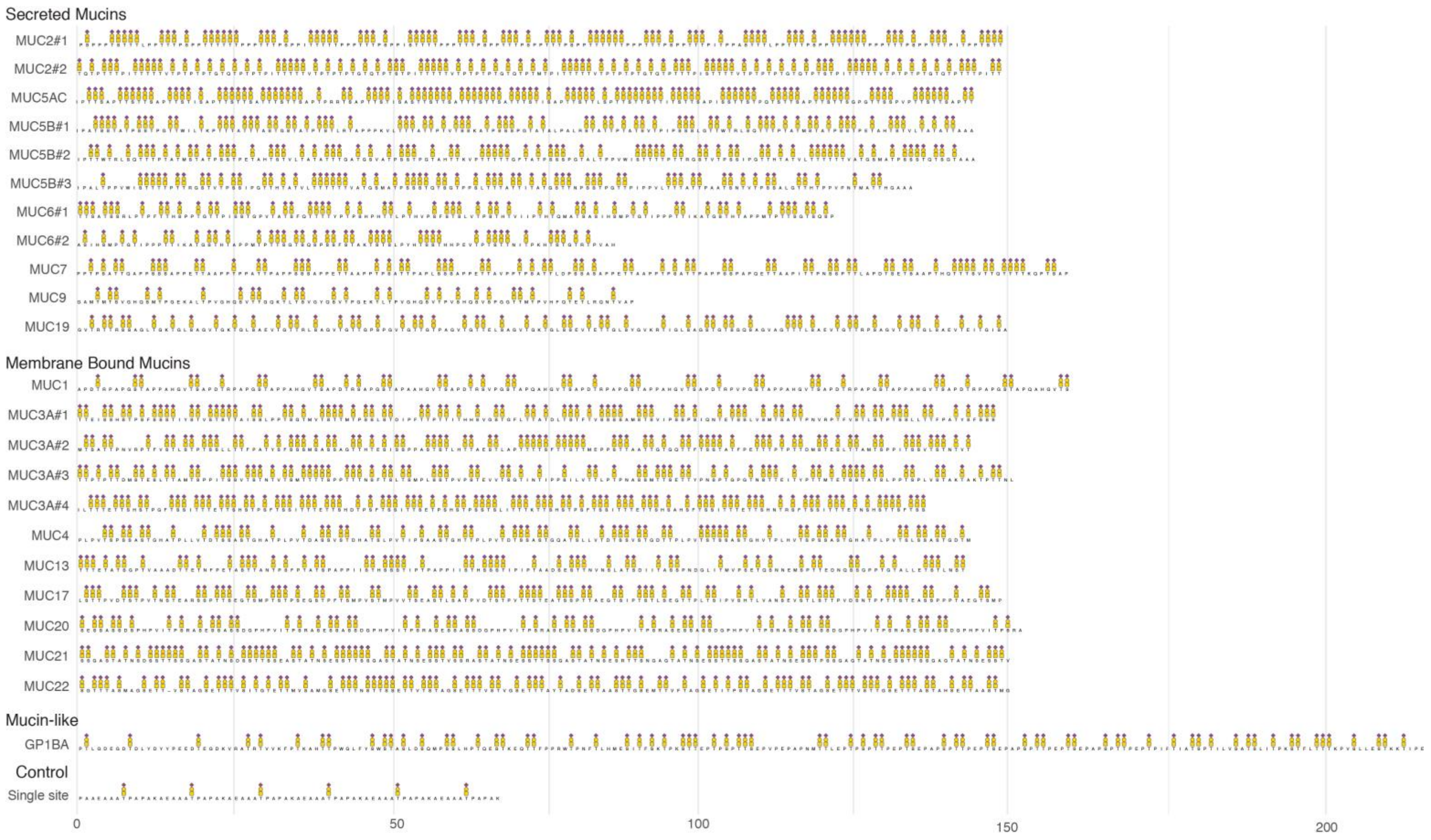

Supplementary Fig. 1a Design of the human mucin TR reporters. Schematic presentation of the imperfect TR amino acid sequences selected for design of the human mucin TR reporters. All Ser/Thr residues are highlighted as potential O-glycosites by glycan symbols (mSTa O-glycans shown for simplicity) to illustrate the characteristic patterning generated with all Ser/Thr residues O-glycosylated.

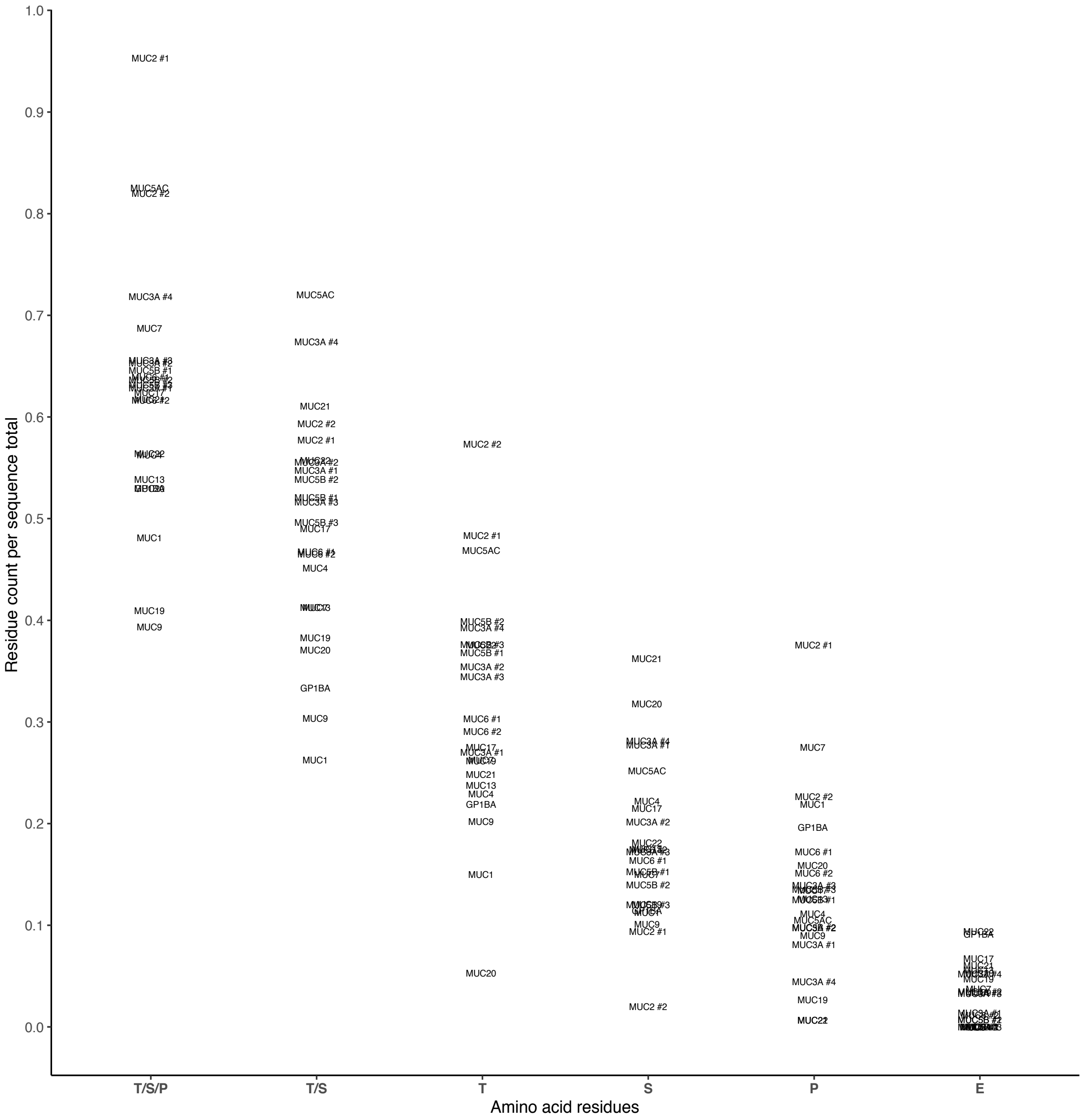

Supplementary Fig. 1b Parallel plot of key amino acid residues in the human mucin TR reporter designs. Residue combinations of TSP, TS and individual T, S, P and E counts in the different TR sequences represented in the mucin TR reporters are shown as residue count per sequence total.

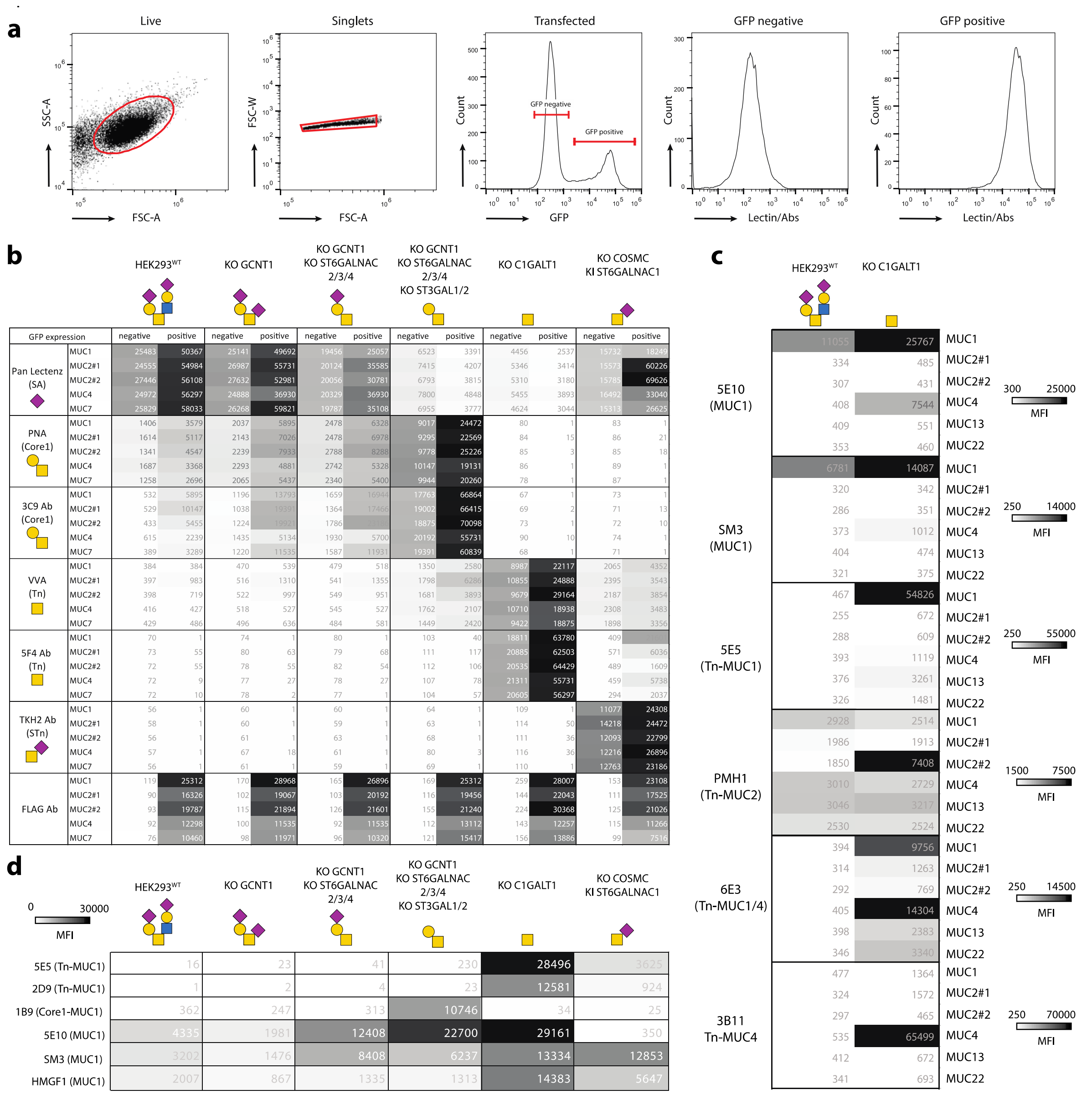

Supplementary Fig. 2 Validation of mucin TR and O-glycoform expression. Analysis of O-glycosylation of the mucin TR reporters expressed in glycoengineered HEK293 cells with antibodies and lectins. **a** Gating strategy to measure lectin or monoclonal antibody binding to HEK293 cells transiently expressing transmembrane mucin-GFP reporters. Live cells were gated on in the side scatter area (SSC-A) versus forward scatter area (FSC-A) plot followed by gating on singlets and cells expressing mucin-GFP reporter (GFP positive) or not expressing mucin-GFP reporter (GFP negative). **b** Flow cytometry analysis of binding of lectins and anti-carbohydrate mAbs to engineered HEK293 cells transiently expressing membrane-bound mucin TR reporters (GFP and FLAG-tagged) as indicated. Primary specificities illustrated with glycan symbols. GFP negative cells (non-transfected) or GFP positive cells (transfected) were analyzed by flow cytometry and mean fluorescent intensity (MFI) values presented as heat map. Surface expression of mucin TR reporters was confirmed by anti-FLAG antibody labelling. **c** Flow cytometry analysis of binding of mucin-specific mAbs to HEK293^{WT} and HEK293$^{KO\ C1GALT1}$ cells transiently expressing mucin TR reporters. **d** Flow cytometry analysis of binding of MUC1 glycoform-specific mAbs to glycoengineered HEK293 cells stably expressing the MUC1 TR reporter. MFI values from representative experiments are shown (greytones indicate high to low MFI values). Source data are provided as a Source Data file.

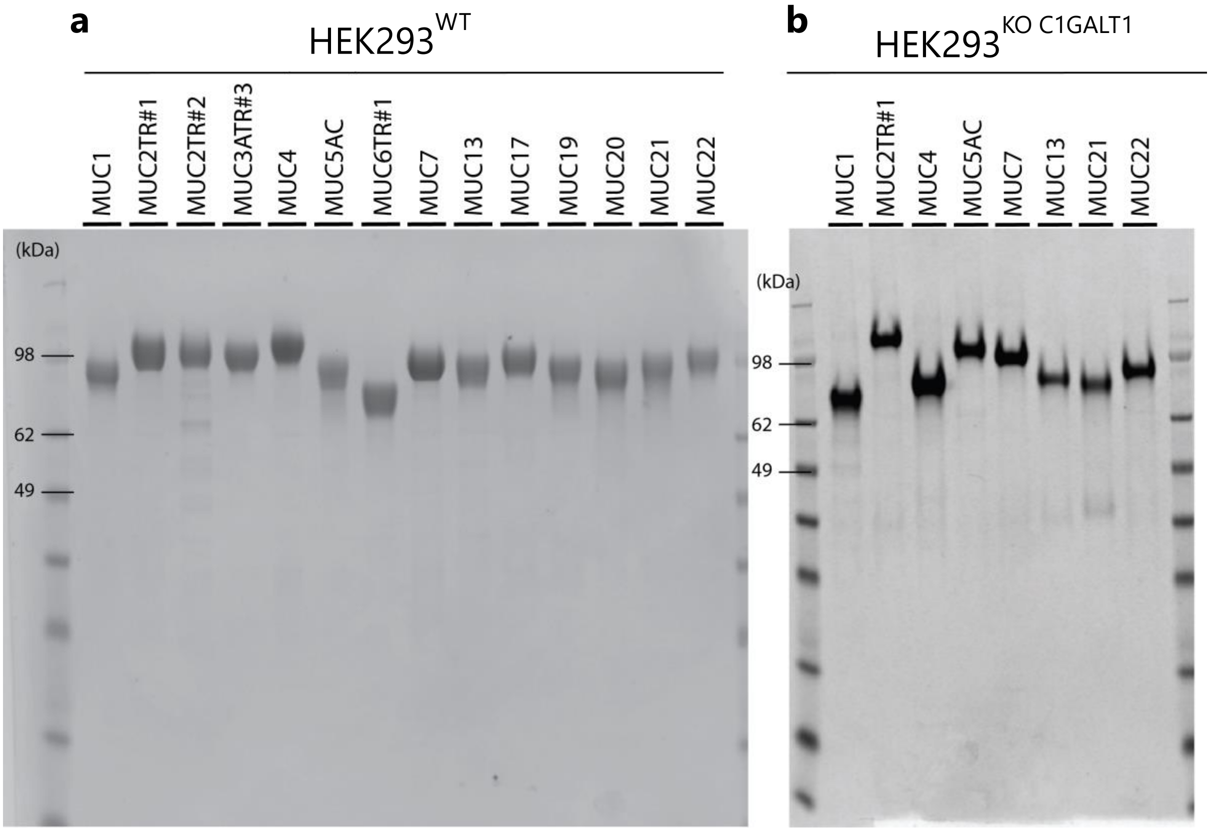

Supplementary Fig. 3 Production of secreted mucin reporter proteins. SDS-PAGE Coomassie analysis of purified secreted mucin TR reporters. **a** Analysis of TR reporters expressed in HEK293^{WT} with heterogeneous core1/2 O-glycans. **b** Analysis of TR reporters expressed in HEK293$^{KO\ C1GALT1}$ with homogenous Tn O-glycans. SDS-PAGE analysis in **a** and **b** were repeated two times with similar results.

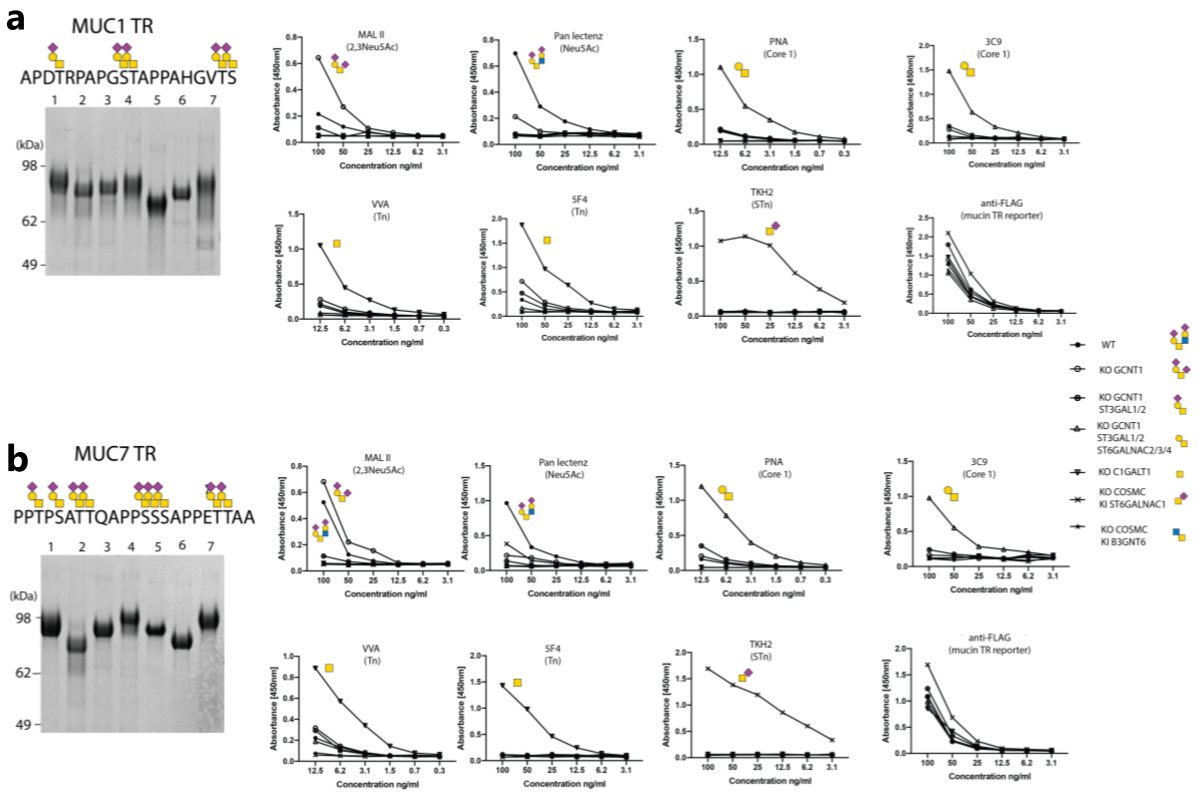

Supplementary Fig. 4 Immunochemical ELISA analysis of glycoengineered MUC1 and MUC7 TR reporters. ELISA analysis of purified secreted MUC1 and MUC7 TR reporters produced in glycoengineered HEK293 cells. **a** SDS-PAGE analysis of MUC1 TR reporters (left) and corresponding ELISA antigen titrations with lectins and anti-carbohydrate mAbs (right) as indicated. Anti-Flag mAb was included to evaluate comparable coating efficiencies. **b** The same analysis with MUC7 TR reporters. Samples loaded for SDS-PAGE analysis corresponded to symbol key (top-to low illustrated right) as follows: Lane 1 HEK293^{WT}, lane 2 HEK293$^{KO\ \mathit{GCNT1}}$, lane 3 HEK293$^{KO\ \mathit{GCNT1/ST3GAL1/2}}$, lane 4 HEK293$^{KO\ \mathit{GCNT1/ST3GAL1/2}\ ST6GALNAC2,3,4}$, lane 5 HEK293$^{KO\ \mathit{C1GALT1}}$, lane 6 HEK293$^{KO\ \mathit{COSMC}\ KI\ ST6GALNAC1}$, lane 7 HEK293$^{KO\ \mathit{COSMC}\ KI\ B3GNT6}$. SDS-PAGE analysis in left panel **a** and **b** were repeated two times with similar results. Source data are provided as a Source Data file.

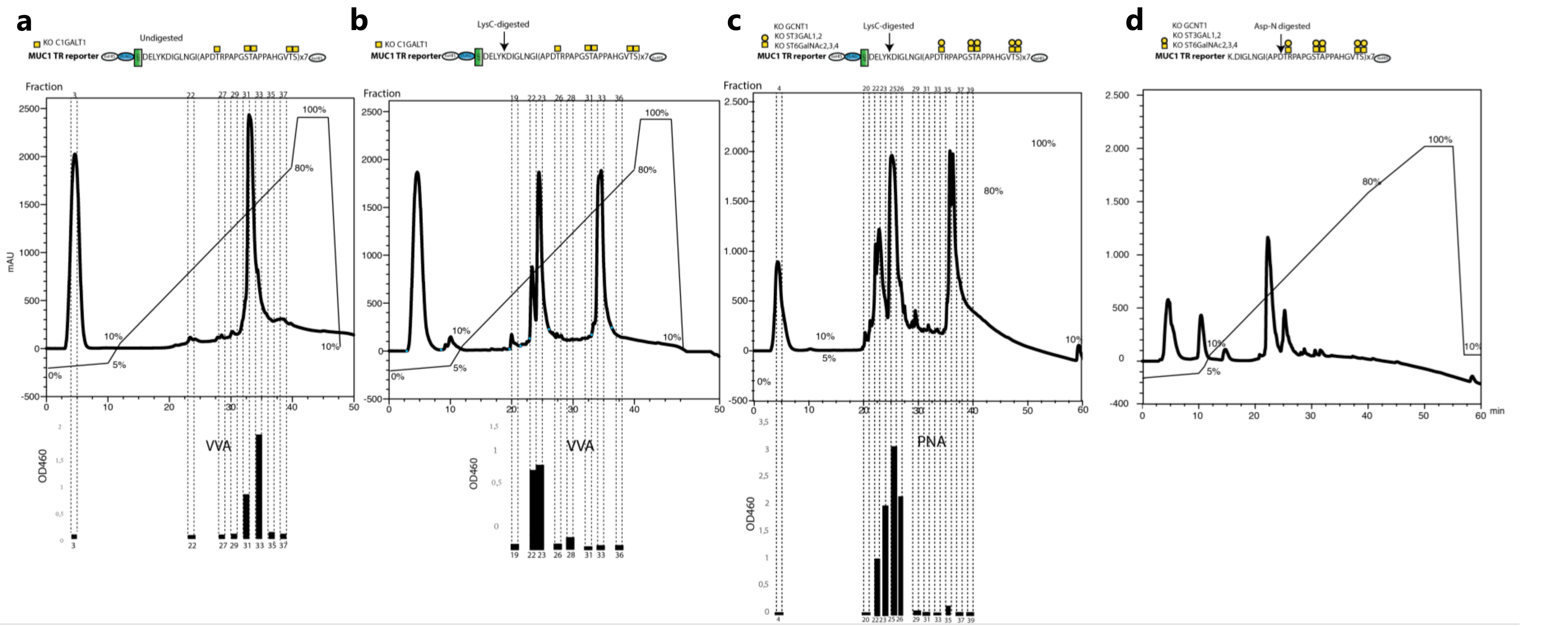

Supplementary Fig. 5 HPLC isolation of MUC1 TR O-glycodomains for intact mass analysis. **a** C4 HPLC isolation of the undigested Tn-glycosylated MUC1 TR reporter with GFP expressed in HEK293$^{KO\ C1GALT1}$ cells. **b** C8 HPLC separation of the corresponding LysC digested TR reporter with the O-glycodomain eluting in fractions 22-23 and the intact GFP module in fraction 33 as verified by VVA lectin ELISA. **c** C4-HPLC of LysC digested T-MUC1 with the O-glycodomain eluting in fractions 22-26 and the intact GFP module in fraction 35 as verified by PNA lectin ELISA, and **d** further digested by AspN. The intact GFP-tagged reporter eluted at ~60% acetonitrile, while the released TR O-glycodomains eluted at ~35% and the digested GFP-tag at ~55%. HPLC data was collected and analyzed with Empower 3 Chromatography Data Software (Waters) and Chromeleon™ 6.8 Chromatography Data System Software (Thermo). Source data are provided as a Source Data file.

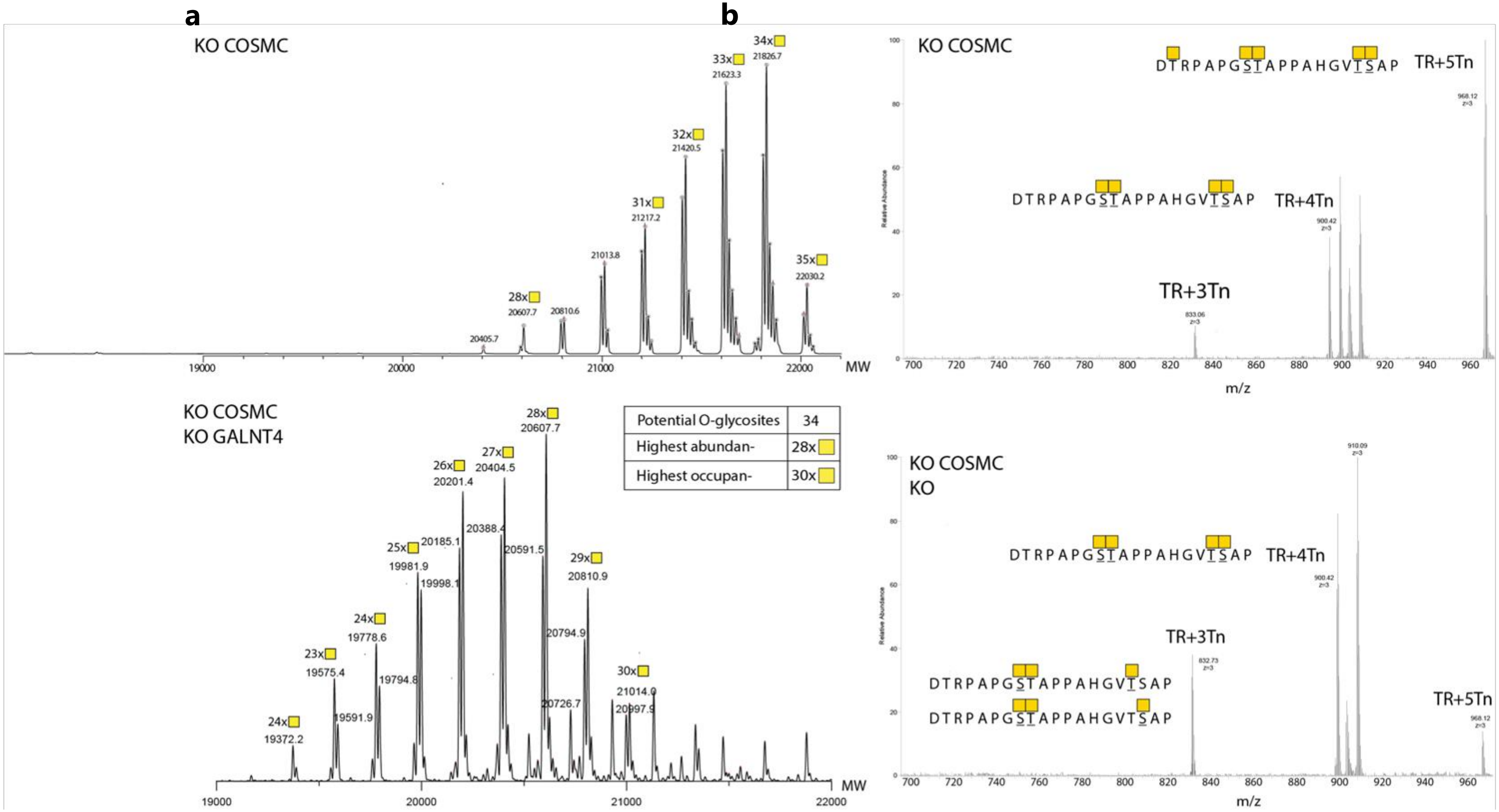

Supplementary Fig. 6 MS analysis of MUC1 TR expressed in GALNT4 KO engineered HEK293$^{KO\ COSMC}$ cells. **a** Deconvoluted intact mass spectra of MUC1 TR O-glycodomains isolated from reporters expressed in HEK293$^{KO\ COSMC}$ (top) and HEK293$^{KO\ COSMC/GALNT4}$ (bottom). **b** Depiction of the three most abundant MUC1-TR glycopeptide precursors applied to MS/MS ETD with identified O-glycosites and predicted O-glycan structures illustrated. MS2 analysis showed that KO of *GALNT4* selectively resulted in loss of O-glycosylation at the glycosite in the PD**T**RP motif. For all intact mass spectra the experimentally determined and theoretically calculated masses are composed in a separate supplementary table 6.

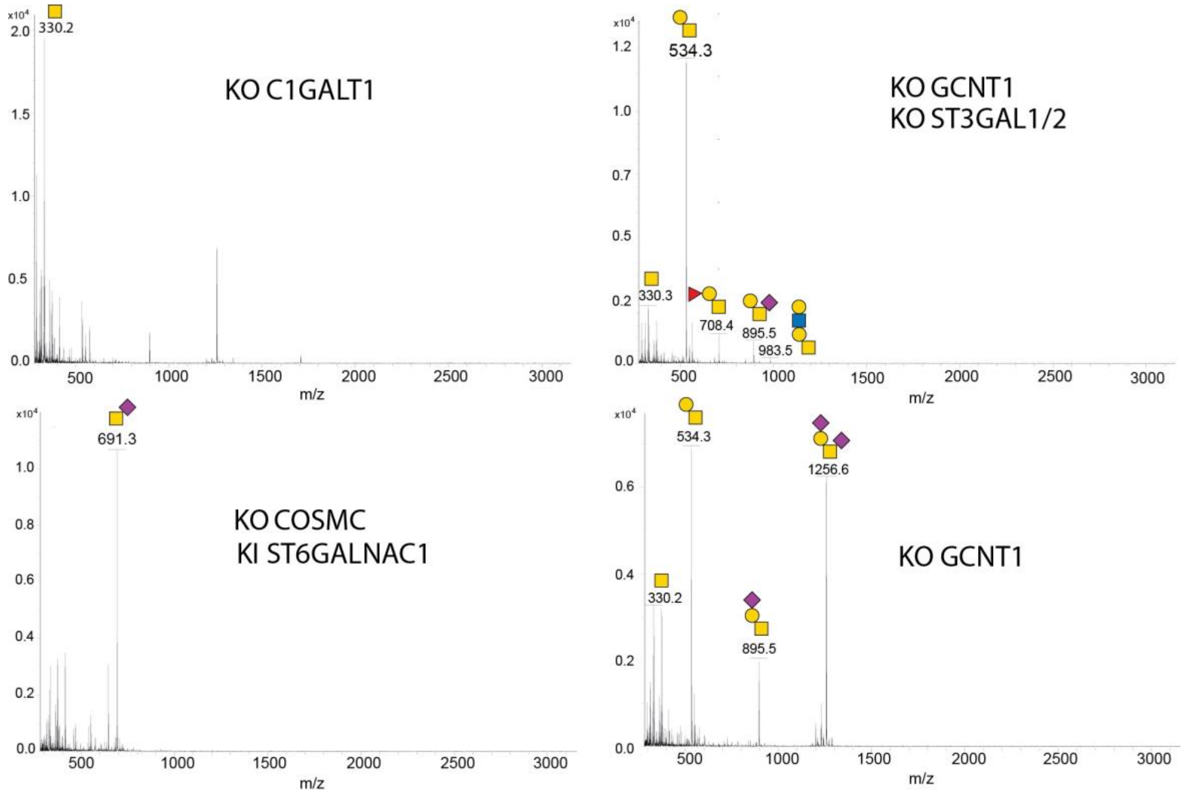

Supplementary Fig. 7 MALDI-TOF profiling of released MUC1 TR O-glycans. MS spectra of O-glycoprofiling of purified mucin TRs produced in HEK293$^{\text{KO C1GALT1}}$, HEK293$^{\text{KO }\textit{COSMC}\text{, KI ST6GALNAC1}}$, HEK293$^{\text{KO GCNT1, ST3GAL1/2}}$ and HEK293$^{\text{KO GCNT1}}$ cells.

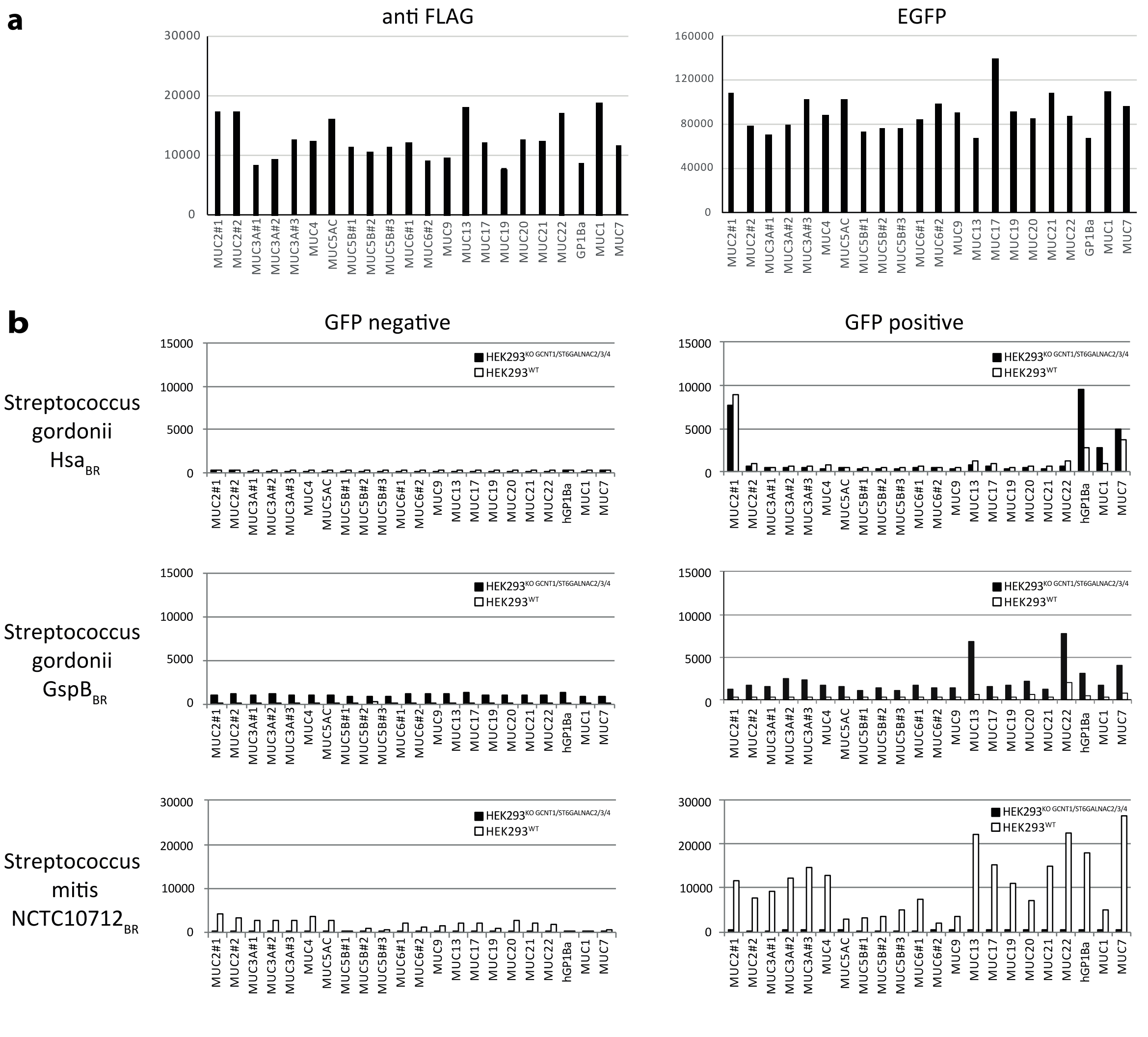

Supplementary Fig. 8 The cell-based mucin display reveals binding specificities of Streptococcus adhesins. a Flow cytometry analysis of surface expression of mucin TR reporters transiently expressed with anti-FLAG mAb and correlation with GFP expression. **b** Bar diagrams showing binding of Siglec-like adhesins from *S. gordonii* (Hsa_{BR} and $GspB_{BR}$) and *S. mitis* ($NCTC10712_{BR}$) to $HEK293^{WT}$ and $HEK293^{KO\ GCNT1/ST6GALNAC2/3/4}$ cells transiently expressing membrane bound mucin TR reporters with GFP. GFP negative (left) and positive (right) cell populations without subtraction are shown. Mean fluorescence intensity (MFI). Representative data of two independent experiments. Source data are provided as a Source Data file.

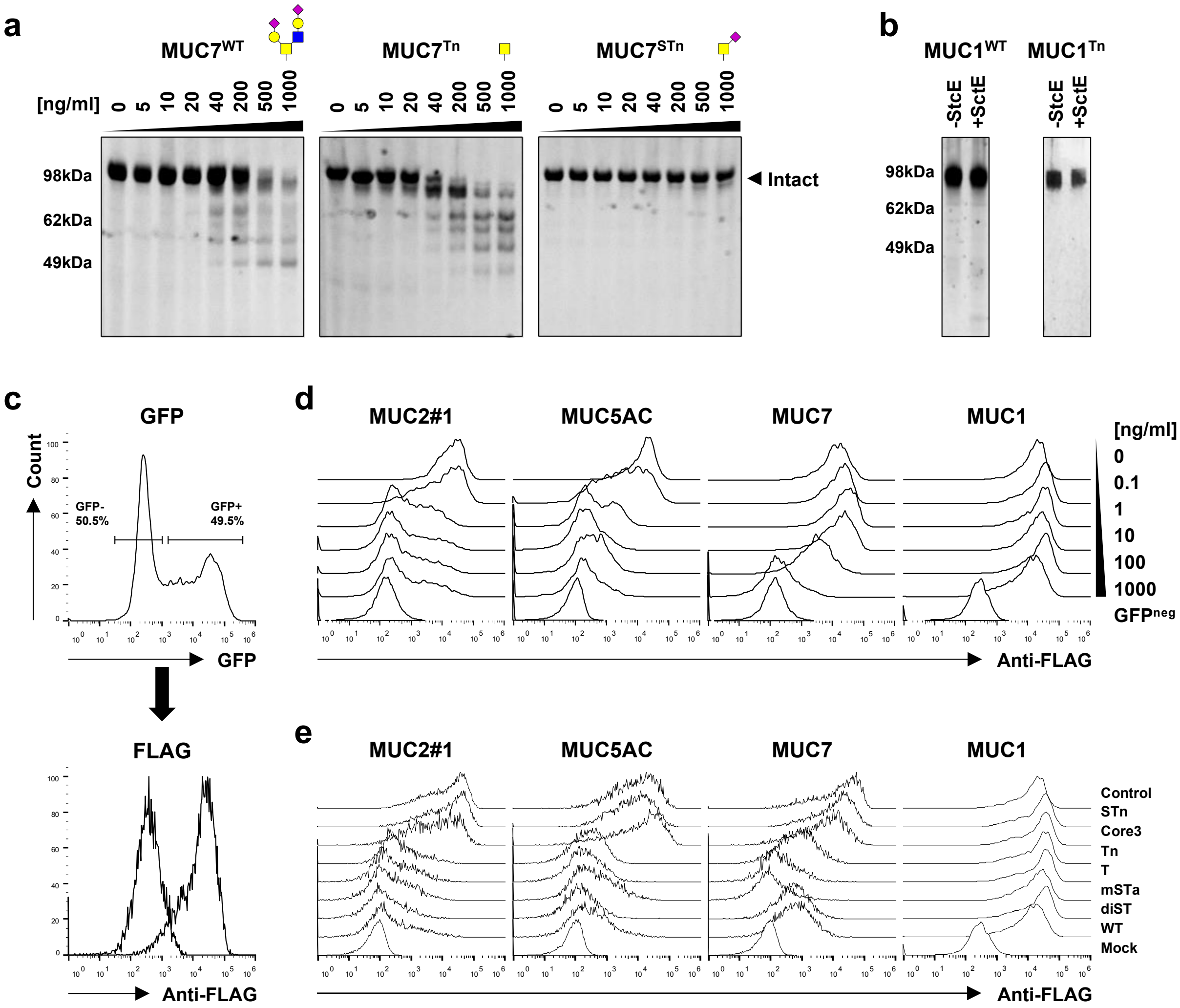

Supplementary Fig. 9 Analysis of StcE on secreted and membrane-bound mucin TR reporters. a SDS-PAGE analysis of StcE (dose titration) digestion of secreted purified MUC7 TR reporters with different glycoforms. **b** SDS-PAGE analysis of StcE digestion of MUC1 TR reporters with 1:10 ratio enzyme to substrate of core2 and Tn glycoforms. **c** Flow cytometry analysis of membrane bound reporters illustrating the gating strategy for transiently expressed GFP-tagged mucin TR reporters in HEK293 cells. Gating for GFP positive cells correlates well with the population of cells labelled by the anti-FLAG mAb detecting surface located mucin TR reporters. **d** Representative histograms of membrane MUC2#1, MUC5AC, MUC7 and MUC1 TR reporters expressed in HEK293^{WT} cells by increasing concentrations of StcE as determined by staining with anti-FLAG mAb. **e** Representative histograms show StcE-mediated cleavage of MUC2#1, MUC5AC, MUC7 and MUC1 TR reporters expressed by HEK293 cells with core2, diST, mSTa, T, Tn, core3 or STn glycosylation. Mock transfected cells and transfected, untreated cells are shown as control. SDS-PAGE analysis in **a** and **b** were repeated with two times with similar results. Source data are provided as a Source Data file.

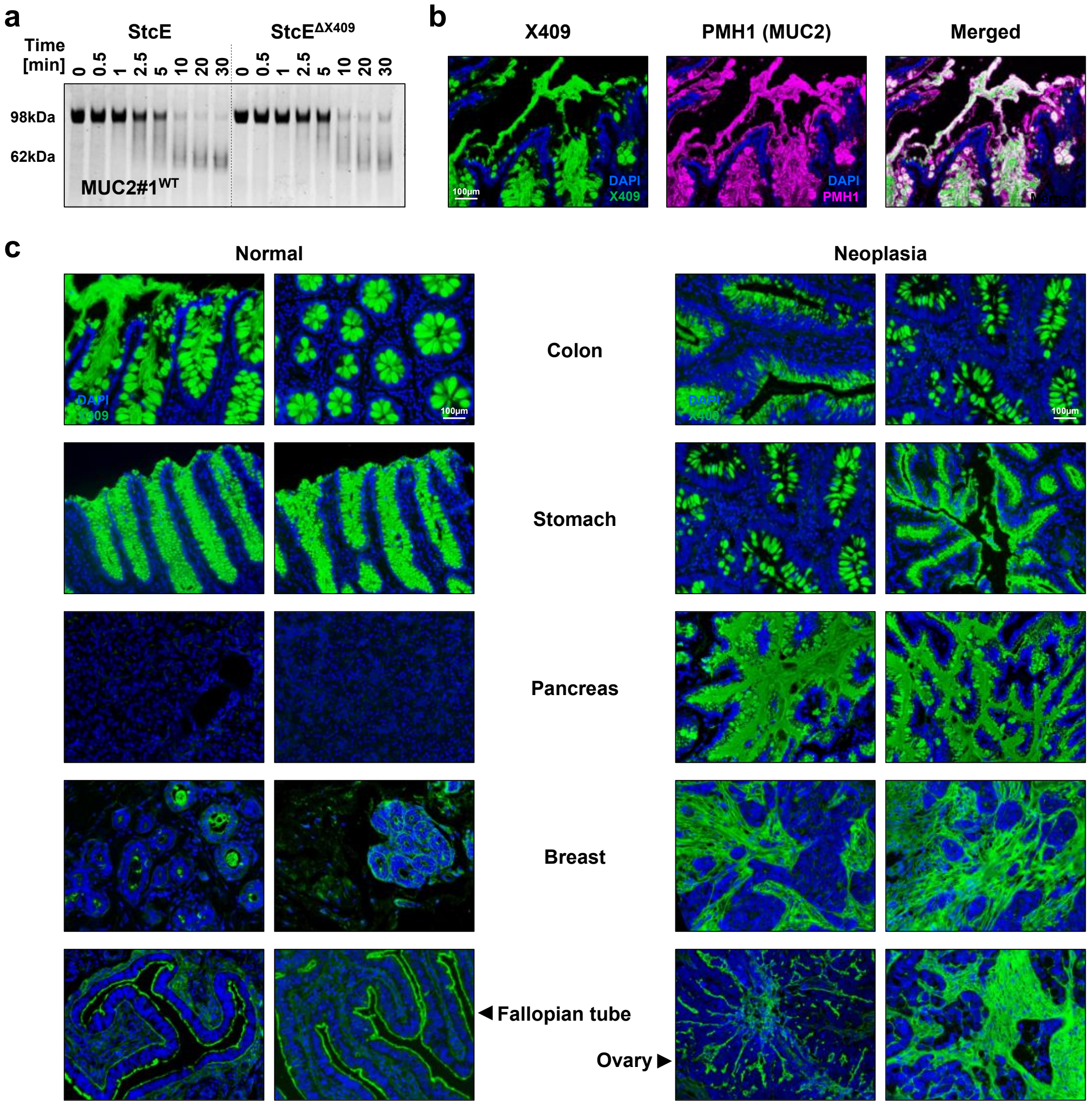

Supplementary Fig. 10 The StcE X409 domain binds mucins *in situ*. **a** SDS-PAGE analysis of StcE and StcE$^{\Delta X409}$ digestion (time course 1:200 ratio) of the MUC2#1 TR reporter expressed in HEK293^{WT}. **b** Representative fluorescence images of sections from normal colon (pretreated with neuraminidase) reacted with X409-GFP and anti-Tn-MUC2 (PMH1) mAb. **c** Images of normal and neoplastic tissue microarray sections reacted with X409-GFP. SDS-PAGE analysis in **a** was repeated multiple times with similar results. Source data are provided as a Source Data file.

Supplementary Table 1. Amino acid sequences of mucin TR reporter constructs used, related to **Figure 1** and **Supplementary Figure 1**.

Gene (HGNC)	Uniprot ID		Position in UniProt ID	length (aa)	Sequence
MUC1	P15941		121-260	140	APDNKPAPGSTAPPAHGVTS APDTRPAPGSTAPPAHGVTS APDTRPAPGSTAPPAHGVTS APDTRPAPGSTAPPAHGVTS APDTRPAPGSTAPPAHGVTS APDTRPAPGSTAPPAHGVTS APDTRPAPGSTAPPAHGVTS
MUC2	Q02817	#1	1406-1554	152	PSPPPTST TTLPPTTT PSPPTTTT TTPPPTTT PSPPITTT TTPPPTTT PSPPISTT TTPPPTTT PSPPTTT<br>PS PPTTT PSPPTTTT TTPPPTTT PS PPTTT PITPPAST TTLPPTTT PSPPTTTT TTPPPTTT PSPPTTT PITPPTST T
		#2	1916-2064	152	PTQTPTTTPITTTTTVTPTPTPT GTQTPTPTPITTTTTVTPTPTPT GTQTPTSTPITTTTTVTPTPTPT GTQTPTMTPITTTTTVTPTPTPT<br>GTQTPTTTPISTTTTVTPTPTPT GTQTPTSTPITTTTTVTPTPTPT GTQTPTTTPITTAA
MUC3A	Q02505	#1	391-538	148	TTEISSHSTPSFSSSTIYSTVSTSTTAISSLPPTSGTMVTSTTMTPSSLSTDIPFTTPTTITHHSVGSTGFLTTATDLTSTFTVSSSSAMSTSVIPSSPSIQNTETSSLVSMTSATTPNVRPTFVSTLSTPTSSLLTTFPATYSFSSS
		#2	502-645	144	MTSATTPNVRPTFVSTLSTPTSSLLTTFPATYSFSSSMSASSAGTTHTESISSPPASTSTLHTTAESTLAPTTTTSFTTSTTMEPPSTTAATTGTGQTTFTSSTATFPETTTPTPTTDMSTESLTTAMTSPPITSSVTSTNTVT
		#3	612-762	151	TTPTPTTDMSTESLTTAMTSPPITSSVTSTNTVTSMTTTTSPPTTTNSFTSLTSMPLSSTPVPSTEVVTSGTINTIPPSILVTTLPTPNASSMTTSETTYPNSPTGPGTNSTTEITYPTTMTETSSTATSLPPTSPLVSTAKTAKTPTTNL
		#4	2227-2361	135	TTTETTSHSTPGFTSS ITTTETTSHSTPSFTSS ITTTETTSHDTPSFTSS ITTSETPSHSTPSSTSL<br>ITTTKTTSHSTPSFTSS ITTTETTSHSAHSFTSS ITTTETTSHNTRSFTSS ITTTETNSHSTTSFTSS
MUC4	E9PDY6		1184-1327	TQT	PLPVTSPSSASTGHAT PLLVTDTSSASTGHAT PLPVTDASSVSTDHAT SLPVTIPSAASTGHTT PLPVTDTSSASTGQAT<br>SLLVTDTSSVSTGDTT PLPVTSTSSASTGHVT PLHVTSPSSASTGHAT PLPVTSLSSASTGDTM
MUC5AC	P98088		2708-2850	142	TTSAPTT STTSAPTT STISAPTT STTSATTT STTSAPTP RRTSAPTT STISASTT STTSATTT STTSATTT<br>STISAPTT STTLSPTT STTSTTIT STTSAPIS STTSTPQT STTSAPTT STTSGPGT TSSPVPTT STTSAPTT
MUC5B	Q9HC84	#1	1889-2029	141	PATSSTATPSSTPGTTWILTKPTTTATTTASTGSTATPTSTLRTAPPPKVLTTTATTPTVTSSKATPSSSPGTATALPALRSTATTPTATSVTPIPSSSLGTTWTRLSQTTTPTATMSTATPSSTPETAHTSTVLTATATT
		#2	1990-2129	140	TTWTRLSQTTTPTATMSTATPSSTPETAHTSTVLTATATTTGATGSVATPSSTPGTAHTTKVPTTTTTGFTATPSSSPGTALTPPVWISTTTTPTTRGSTVTPSSIPGTTHTATVLTTTTTTVATGSMATPSSSTQTSGT
		#3	2070-2199	130	ALTPPVWISTTTTPTTRGSTVTPSSIPGTTHTATVLTTTTTTVATGSMATPSSSTQTSGTPPSLTTTATTITATGSTTNPSSTPGTTPIPPVLTTTATTPAATSNTVTPSSALGTTHTPPVPNTMATTHG
MUC6	Q6W4X9	#1	1786-1907	123	TSATSSRLPTPFTTHSPPTGTTPISSTGPVTATSFQTTTTYPTPSHPHTTLPTHVPSFSTSLVTPSTHTVIIPTHTQMATSASIHSMPTGTIPPPTTIKATGSTHTAPPMTPTTSGTSQSPS
		#2	1868-1953	86	SIHSMPTGTIPPPTTIKATGSTHTAPPMTPTTSGTSQSPSSFSTAKTSTSLPYHTSSTHHPEVTPTSTTNITPKHTSTGTRTPVAH
MUC7	Q8TAX7		192-351	160	PPTPSATTQAPPSSSAPPE TTAAPPTPPATTPAPPSSSAPPE TTAAPPTPSATTPAPLSSSAPPE TTAVPPTPSATTLDPSSASAPPE<br>TTAAPPTPSATTPAPPSSPAPQE TTAAPITTPNSSPTTLAPDTSET SAAPTHQTTTSVTTQTTTTKQPTSAP
OVGP1 (MUC9)	Q12889		476-564	89	AMTMTSVGHQSMTP GEKALTPVGHQSVTT GQKTLTSVGYQSVTP GEKTLTPVGHQSVTP VSHQSVSPGGTTMTP VHFQTETLRQNTVAP
MUC13	Q9H3R2		30-171	142	TTETATSGPTVAAADTTETNFPE TASTTANTPSFPTATS PAPPIISTHSSSTIPT PAPPIISTHSSSTIPI PTAADSESTTNVNSLA<br>TSDIITASSPNDGLIT MVPSETQSNNEMSPTT EDNQSSGPPTGTALLE TSTLNST
MUC17	Q685J3		2181-2329	149	LSTTPVDTSTPVTNSTEARSSPTTSEGTSMPTSTPSEGSTPFTSMPVSTMPVVTSEAST LSATPVDTSTPVTTSTEATSSPTTAEGTSIPTSTLSEGTTPLTSIPVSHTLVANSEVST<br>LSTTPVDSNTPFTTSTEASSPPPTAEGTSMP
MUC19	Q7Z5P9		3353-3501	149	VTRTTRSSA GLTGKTGLSA GVTGKTGLSA EVTGTTRLSA GVTGTTGPSP GVTGTTGTPA GVTGTTELSA GVTGKTGLSS<br>EVTETTGLSY GVKRTIGLSA GSTGTSGQSA GVAGTTTLSA EVTGTTRPSA GVTGTTGLSA EVTEITGISA
MUC20	Q8N307		175-325	151	ESSASSDSPHPVITPSRA SESSASSDGPHPVITPSRA SESSASSDGPHPVITPSRA SESSASSDGPHPVITPSRA<br>SESSASSDGPHPVITPSRA SESSASSDGPHPVITPSRA SESSASSDGPHPVITPSRA SESSASSDGPHPVITPSRA
MUC21	Q5SSG8		130-278	149	SGASTATNSDSSTT SSGASTATNSDSSTT SSEASTATNSESSTT SSGASTATNSESSTV SSRASTATNSESSTT<br>SSGASTATNSESRTT SNGAGTATNSESSTT SSGASTATNSESSTP SSGAGTATNSESSTT SSGAGTATNSESSTV
MUC22	E2RYF6		336-483	148	GTTTASMAG SETTVSTAG SETTTVSITG TETTMVSAMG SETTTNSTTS SETTVTSTAG SETTTVSTVG SETTTAYTAD<br>SETTAASTTG SEMTTVFTAG SETITPSTAG SETTTVSTAG SETTTVSTTG SETTTASTAH SETTAASTMG
GP1BA	P07359		281-499	219	PTLGDEGDTDLYDYYPEEDTEGDKVRATRTVVKFPTKAHTTPWGLFYSWSTASLDSQMPSSLHPTQESTKEQTTFPPRWTPNFTLHMESIT FSKTPKSTTEPTP SPTTSEPVPEPAP NMTTLEPTP SPTTPEPTSEPAP<br>SPTTPEPTSEPAP SPTTPEPTSEPAP SPTTPEPTPIPTI ATSPTILVSATSLITPKSTFLTTTKPVSLLESTKKTIPELD
Ctrl	-		-	67	PAEAAATPAPAK AEAAATPAPAK AEAAATPAPAK AEAAATPAPAK AEAAATPAPAK AEAAATPAPAK

Supplementary Table 2. Summary of engineered HEK293 isogenic cell library generated to date, related to **Figure 1**.

HEK293 engineered cells		In-dels#1		In-dels#2		In-dels#3		In-dels#4		In-dels#5		In-dels#6
ΔCOSMC<br>(Tn)	Cosmc	WT :ATAGAATGCA ccaCCAT-GAGCAT<br>+1 :ATAGAATGCA ccaCCATTGAGCAT										
ΔC1GALT1<br>(Tn)	C1GALT1	WT :GGCTACAT-GAG tggAGGAGCAGGA<br>+1 :GGCTACATTGAGtggAGGAGCAGGA										
ΔGCNT1<br>(ΔCore2)	GCNT1	WT :TAAAAAGCGC cctCGG-TGGACAC<br>-5 :TAAAAAGCGC c------TGGACAC<br>+1 :TAAAAAGCGC cctCGGTTGGACAC										
ΔCOSMC<br>KI B3GnT6<br>(Core3)	B3GNT6	Target KI confirmed<br>by Junction PCR	Cosmc	WT :ATAGAATGCA ccaCCAT-GAGCAT<br>+1 :ATAGAATGCA ccaCCATTGAGCAT								
ΔCOSMC<br>KI ST6GalNAc1<br>(STn)	ST6GALNAC1	Target KI confirmed<br>by Junction PCR	Cosmc	WT :ATAGAATGCA ccaCCAT-GAGCAT<br>+1 :ATAGAATGCA ccaCCATTGAGCAT								
ΔST3GAL1/2<br>ΔGCNT1<br>(mSTb)	ST3GAL1	WT :GATCCTGGTG cccTTC--AAGACCA<br>+1 :GATCCTGGTG cccTTC-AAAGACCA<br>+2 :GATCCTGGTG cccTTCAAAAGACCA	ST3GAL2	WT :GATGCCGGTG cctCCG-ACTGGTT<br>-2 :GATGCCGGTG cctCCGA--TGGTT<br>+1 :GATGCCGGTG cctCCGAACTGGTT	GCNT1	WT :TAAAAAGCGC cctCGG-TGGACAC<br>-5 :TAAAAAGCGC c------TGGACAC<br>+1 :TAAAAAGCGC cctCGGTTGGACAC						
ΔGCNT1<br>Δ ST6GALNAC2/3/4<br>(mSTa)	GCNT1	WT :TAAAAAGCGC cctCGG-TGGACAC<br>-5 :TAAAAAGCGC c------TGGACAC<br>+1 :TAAAAAGCGC cctCGGTTGGACAC	ST6GALNAC2	WT :CAACACAAAG cccCGTA-TGGCTG<br>+1 :CAACACAAAG cccCGTAATGGCTG	ST6GALNAC3	WT :TACAGGCGGC cccTTC-GAACTCA<br>+1 :TACAGGCGGC cccTTCGGAACTCA<br>+1 :TACAGGCGGC cccTTCCGAACTCA	ST6GALNAC4	WT :CAGCGCAGCA cccTGCG-TGTCGT<br>+1 :CAGCGCAGCA cccTGCGGTGTCGT				
ΔGCNT1<br>ΔST6GALNAC2/3/4<br>ΔST3GAL1/2 (Core1)	GCNT1	WT :TAAAAAGCGC cctCGG-TGGACAC<br>-5 :TAAAAAGCGC c------TGGACAC<br>+1 :TAAAAAGCGC cctCGGTTGGACAC	ST6GALNAC2	WT :CAACACAAAG cccCGTA-TGGCTG<br>+1 :CAACACAAAG cccCGTAATGGCTG	ST6GALNAC3	WT :TACAGGCGGC cccTTC-GAACTCA<br>+1 :TACAGGCGGC cccTTCGGAACTCA<br>+1 :TACAGGCGGC cccTTCCGAACTCA	ST6GALNAC4	WT :CAGCGCAGCA cccTGCG-TGTCGT<br>+1 :CAGCGCAGCA cccTGCGGTGTCGT	ST3GAL1	WT :GATCCTGGTG cccTTC-AAGACCA<br>+1 :GATCCTGGTG cccTTCAAAGACCA	ST3GAL2	WT :GATGCCGGTG cctCCG-ACTGGTT<br>-4 :GATGCCGGTG cctCCGA----GTT<br>+1 :GATGCCGGTG cctCCGAACTGGTT
ΔGALNT4	GALNT4	WT :TATATC-TTCG tggAGCTCTTGGT<br>+1 :TATATCTTTCGtggAGCTCTTGGT										
ΔGALNT7/10	GALNT7	WT :ACAGATTCAAA cctGTGGTACCAT<br>-1 :ACAGATTCAAA cctGTG-TACCAT	GALNT10	WT :GAAGACCTTA cccCATG-ACCGATG<br>+1 :GAAGACCTTA cccCATGGACCGATG								
ΔGALNT1/2/3	GALNT1	WT :TTCCTGGATG cccATT-GTGAGTGTA<br>-8 :TTCCTGGATG cccATT---------A<br>+1 :TTCCTGGATG cccATTGGTGAGTGTA	GALNT2	WT :CTGCCGGCCA ccaGCG--TGGTGATC<br>+2 :CTGCCGGCCA ccaGCGGTTGGTGATC<br>-1: CTGCCGGCCA ccaG-G--TGGTGATC	GALNT3	WT :ACCATAACCG tggAAA-TTTTGACT<br>+1: ACCATAACCG tggAAAATTTTGACT<br>-1: ACCATAACCGtggAAA--TTTGACT						
ΔCosmc, GALNT4	Cosmc	WT :ATAGAATGCA ccaCCAT-GAGCAT<br>+1 :ATAGAATGCA ccaCCATTGAGCAT	GALNT4	WT :TATATC-TTCG tggAGCTCTTGGT<br>+1 :TATATCTTTCGtggAGCTCTTGGT								
ΔCosmc, GALNT7/10	C1GALT1	WT :GGCTACAT-GAG tggAGGAGCAGGA<br>+1 :GGCTACATTGAGtggAGGAGCAGGA	GALNT7	WT :TGGACTAGCT cctGGGG-AGGACA<br>+1 :TGGACTAGCT cctGGGGAAGGACA<br>-2: TGGACTAGCT --tGGGG-AGGACA	GALNT10	WT :GAAGACCTTA cccCATG-ACCGATG<br>+1 :GAAGACCTTA cccCATGGACCGATG						
ΔC1GALT1<br>ΔGALNT1/2/3	C1GALT1	WT :GGCTACAT-GAG tggAGGAGCAGGA<br>+1 :GGCTACATTGAGtggAGGAGCAGGA<br>+1 :GGCTACATGGAGtggAGGAGCAGGA	GALNT1	WT :TTCCTGGATG cccATT-GTGAGTGTA<br>-8 :TTCCTGGATG cccATT---------A<br>+1 :TTCCTGGATG cccATTGGTGAGTGTA	GALNT2	WT :CTGCCGGCCA ccaGCG--TGGTGATC<br>+2 :CTGCCGGCCA ccaGCGGTTGGTGATC<br>-1: CTGCCGGCCA ccaG-G--TGGTGATC	GALNT3	WT :ACCATAACCG tggAAA-TTTTGACT<br>+1: ACCATAACCG tggAAAATTTTGACT<br>-1: ACCATAACCGtggAAA--TTTGACT				

Note: Nucleic acids in RED are the insertion or deletion
Nucleic acids in Blue are the PAM sequence

Supplementary Table 3. List of CRISPR gRNA design and PCR primers used in this study.

Gene	gRNA	Forward primer (5'-3')	Reverse primer (5'-3')
Cosmc	GTAGGTGATGATGCTCATGG	TGAAGGGTGTGATGCTTGGAA	ACTGCAGCCCAAAGACTCAC
C1GALT1	GTAAAGCAGGGCTACATGAG	CCTGCTGTGGGACTGAAAAC	TGCATCTCCCCAGTGCTAAG
GCNT1	TAGTCGTCAGGTGTCCACCG	GACACTTGGAGCTTGCTGG	GGCATATAGATGGCCCTCAGC
ST3GAL1	TCCAAGTCGATGGTCTTGAA	GTGCCCGTGCTACAAGACTC	AGTTGGAGTAGTCGGGGAGG
ST3GAL2	GTGGCTGTCAAACCAGTCGG	TGCCAGAATAGGCAGGCTAC	TGTTACCGTCAAAGTGGCTG
ST6GALNAC2	GAGCCCCCGCCAGCCATACG	CTCAGCCCTCACCTTCTCAC	ATCACCAGTGCTATGAGGGC
ST6GALNAC3	GTATCCATAGTGAGTTCGAA	TCCTTCTGTGACTGCCTTTGG	TTCAGTGAGTTGGAAGCCCTC
ST6GALNAC4	TGTGTGTGAGACGACACGCA	CTCTCTGTCTCTTTCTCCCTGC	GGGCCTTCTGGAAGTAGTGTG
GALNT1	TCCCACTGTACACTCACAAT	GAATAGTGCCAGGCCACACT	AAAGCAAACTTGGGAGGAAAT
GALNT2	GTGAAACGTGATCACCACGC	CCATCCCAGTTGGTCAGTCT	CTGTGCTGAGCAGTCAGGAG
GALNT3	TATGGAAGTAACCATAACCG	TCCCTCCAGGTGAGTGTTTC	AAAGCAAACAGTGTGTACATATTCAA
GALNT4	AACAGTGGCCTATATCTTCG	CTGCTGGGAAGTACCTGAGC	TCCTCGTTGAGCTGGAGTTT
GALNT7	ATGCCCAACCGAGGCGGCAA	TTAATGGCCCGCTTGTATTC	CGAAGCACAGGATCATGGTA
GALNT10	CTCTCTCAGCATCGGTCATG	GCTTGCTCCCCTCCTACTCT	ACAACAGCCAGGGAAACATC

Supplementary Table 4. A peak list of intact mass spectra experimentally determined (MW ex) and theoretically calculated (MW cal) masses related Figure 2a.

MUC1 KO *C1GALT1*			MUC1 KO *GCNT1*, KO *ST3GAL1/2*			MUC1 KO *GCNT1*, KO *ST3GAL1/2*			MUC1 KO *GCNT1*		
Number of HexNAc	MW ex	MW cal	Number of HexNAc	MW ex	MW cal	Number of HexNAc	MW ex	MW cal	Number of HexNAc	MW ex	MW cal
28*	20607.7	20607.5	18	18560.1	18559.6	28	25132.5	25131.5	27	24766.1	24766.1
29*	20810.6	20810.7	19	18764.0	18762.8	29	25497.8	25496.8	28	25130.9	25131.5
30*	21013.8	21013.9	20	18966.9	18966.0	30	25861.9	25862.1	29	25497.2	25496.8
31*	21217.2	21217.1	21	19170.1	19169.2	31	26228.3	26227.5	30	25861.5	25862.1
32*	21420.5	21420.3	22	19372.5	19372.4	32	26593.6	26592.8	31	26227.7	26227.5
33*	21623.3	21623.5	23	19575.8	19575.6	33	26958.8	26958.1	32	26592.9	26592.8
34*	21826.7	21826.7	24	19779.0	19778.8	34	27323.9	27323.5	33	26958.3	26958.1
35*	22030.2	22029.9	25	19982.0	19982.0	35	27689.6	27688.8	34	27323.5	27323.5
			26	20185.5	20185.1	36	28054.8	28054.1			
			27	20388.9	20388.3						
			28	20592.8	20591.5						
			29	20795.9	20794.7						
			30	20999.0	20997.9						
			31	21201.1	21201.1						
			32	21404.4	21404.3						
			33	21607.7	21607.5						
			34	21810.8	21810.7						
			35	22014.7	22013.9						

*) Oxidated

Supplementary Table 5. A peak list of intact mass spectra experimentally determined (MW ex) and theoretically calculated (MW cal) masses related Figure 3.

MUC2#1 KO *C1GALT1*			MUC2#2 KO *C1GALT1*			MUC5 KO *C1GALT1*			MUC7 KO *C1GALT1*			MUC13 KO *C1GALT1*			MUC22 KO *C1GALT1*		
Number of HexNAc	MW ex	MW cal	Number of HexNAc	MW ex	MW cal	Number of HexNAc	MW ex	MW cal	Number of HexNAc	MW ex	MW cal	Number of HexNAc	MW ex	MW cal	Number of HexNAc	MW ex	MW cal
73	31256.8	31257.2	85	34024.5	34023.8	97	35167.3	35166.0	57	28932.8	28932.7	48**	25792.1	25790.4	59**	28071.1	28071.2
76	31866.0	31866.7	86	34227.5	34227.0	98	35370.3	35369.2	58	29135.0	29135.9	49**	25995.2	25993.6	63**	28884.3	28883.9
77	32069.7	32069.9	87	34430.7	34430.2	99	35573.7	35572.4	59	29338.4	29339.1	50**	26198.3	26196.8	68***	29918.0	29915.9
78	32272.6	32273.1	88	34633.7	34633.4	100	35776.8	35775.6	60	29541.7	29542.3	51**	26401.3	26400.0	69***	30121.0	30119.1
79	32476.0	32476.3	89	34836.7	34836.6	101	35979.9	35978.8	61	29745.0	29745.5	52**	26604.3	26603.2	70***	30324.2	30322.3
80*	32696.8	32695.5	90	35039.9	35039.8	102	36182.8	36182.0	62	29948.2	29948.6	53**	26807.3	26806.4	71***	30527.2	30525.5
81*	32900.0	32898.7				103	36385.6	36385.1	63	30151.7	30151.8	54**	27010.7	27009.6	72***	30729.7	30728.7
82*	33103.0	33101.9				104	36589.2	36588.3	64	30354.9	30355.0	55**	27213.3	27212.8	73***	30933.3	30931.9
83*	33306.2	33305.1				105	36791.9	36791.5	65	30558.3	30558.2				74***	31136.3	31135.0
84*	33509.3	33508.3							66	30761.1	30761.4				75***	31339.8	31338.2
85*	33712.9	33711.5							67	30964.7	30964.6						
86*	33915.7	33914.7															

*) Oxidated

**) Deamidated after PNGaseF treatment (1xN-glyco site)

***) Deamidated after PNGaseF treatment (1xN-glyco site)+Oxidation

Supplementary Table 6. A peak list of intact mass spectra experimentally determined (MW ex) and theoretically calculated (MW cal) masses related Supplementary Figure 7a.

MUC1 KO C1GALT1			MUC1 KO C1GALT1/GALNT4		
Number of HexNAc	MW ex	MW cal	Number of HexNAc	MW ex	MW cal
28*	20607.7	20607.5	22	19372.3	19372.4
29*	20810.6	20810.7	23	19575.2	19575.6
30*	21013.8	21013.9	23*	19592.1	19591.6
31*	21217.2	21217.1	24	19778.9	19778.8
32*	21420.5	21420.3	24*	19794.7	19794.8
33*	21623.3	21623.5	25	19981.6	19982.0
34*	21826.7	21826.7	25*	19998.1	19998.0
35*	22030.2	22029.9	26	20185.0	20185.2
			26*	20201.6	20201.1
			27	20388.3	20388.3
			27*	20404.1	20404.3
			28	20591.3	20591.5
			28*	20607.4	20607.5
			29	20795.2	20794.7
			29*	20810.9	20810.7
			30	20997.5	20997.9
			30*	21014.3	21013.9

*) Oxidated

**) Deamidated after PNGaseF treatment (1xN-glyco site)

***) Deamidated after PNGaseF treatment (1xN-glyco site)+Oxidation
