# Supplementary material for: Display of the human mucinome with defined O-glycans by gene engineered cells
Source: Nat Commun. 2021 Jul 1;12:4070. doi: 10.1038/s41467-021-24366-4 (PMC8249670; doi:10.1038/s41467-021-24366-4)
Supplement: Supplementary file 3 — Reporting Summary [file 41467_2021_24366_MOESM3_ESM.pdf]

nature research

Corresponding author(s): Henrik Clausen

Last updated by author(s): Jun 1, 2021

Reporting Summary

Nature Research wishes to improve the reproducibility of the work that we publish. This form provides structure for consistency and transparency in reporting. For further information on Nature Research policies, see our Editorial Policies and the Editorial Policy Checklist.

Statistics

For all statistical analyses, confirm that the following items are present in the figure legend, table legend, main text, or Methods section.

n/a	Confirmed	
☐	☒	The exact sample size (n) for each experimental group/condition, given as a discrete number and unit of measurement
☐	☒	A statement on whether measurements were taken from distinct samples or whether the same sample was measured repeatedly
☒	☐	The statistical test(s) used AND whether they are one- or two-sided<br>*Only common tests should be described solely by name; describe more complex techniques in the Methods section.*
☒	☐	A description of all covariates tested
☒	☐	A description of any assumptions or corrections, such as tests of normality and adjustment for multiple comparisons
☐	☒	A full description of the statistical parameters including central tendency (e.g. means) or other basic estimates (e.g. regression coefficient) AND variation (e.g. standard deviation) or associated estimates of uncertainty (e.g. confidence intervals)
☒	☐	For null hypothesis testing, the test statistic (e.g. F, t, r) with confidence intervals, effect sizes, degrees of freedom and P value noted<br>*Give P values as exact values whenever suitable.*
☒	☐	For Bayesian analysis, information on the choice of priors and Markov chain Monte Carlo settings
☒	☐	For hierarchical and complex designs, identification of the appropriate level for tests and full reporting of outcomes
☒	☐	Estimates of effect sizes (e.g. Cohen's d, Pearson's r), indicating how they were calculated

Our web collection on statistics for biologists contains articles on many of the points above.

Software and code

Policy information about availability of computer code

Data collection	HPLC data were collected with Empower 3 Chromatography Data Software (Waters), in which data analysis was also performed. In flow cytometry experiments, we have employed the Sony SONY SA3800 software for data collection. For immunohistochemistry a Zeiss microscopic system was used and protein gels were imaged with an ImageQuant LAS 4000 system (GE Healthcare). Mass spectrometry experiments were collected with OrbiTrap Fusion/Lumos (Thermo) and Autoflex (Bruker).
Data analysis	We used Chromeleon™ 6.8 Chromatography Data System Software (Thermo) for HPLC data analysis, FlowJo X 10.0.7r2 for flow cytometry , ImageJ (NIH) Version 2.1.0/1.53c for immunohistochemistry and ImageQuant software (GE Healthcare) for protein gels, FlexAnalysis (Bruker), BioPharma Finder Verison 3.2/4.0 (demo) (Thermo), and SysBioWare software.(Vakhrushev, S., Dadimov, D. & Peter-Katalinic, J. Software platform for high-throughput glycomics. Analytical chemistry 81, 3252-3260 (2009)) for mass spectrometry data analysis.

For manuscripts utilizing custom algorithms or software that are central to the research but not yet described in published literature, software must be made available to editors and reviewers. We strongly encourage code deposition in a community repository (e.g. GitHub). See the Nature Research guidelines for submitting code & software for further information.

Data

Policy information about availability of data

All manuscripts must include a data availability statement. This statement should provide the following information, where applicable:

- Accession codes, unique identifiers, or web links for publicly available datasets
- A list of figures that have associated raw data
- A description of any restrictions on data availability

All data generated or analyzed during this study are included in this article and supplementary information files. The mass spectrometry proteomics data has been deposited to the ProteomeXchange Consortium via the PRIDE repository with the dataset identifier PXD024851 [https://www.ebi.ac.uk/pride/archive/projects/

PXD024851]. The source data underlying Figures 4a, 5b-d, 6b-e, 7 and Supplementary Figures 2, 4, 5a-c, 8, 9 and 10a are provided as a Source Data file.

Field-specific reporting

Please select the one below that is the best fit for your research. If you are not sure, read the appropriate sections before making your selection.

☒ Life sciences ☐ Behavioural & social sciences ☐ Ecological, evolutionary & environmental sciences

For a reference copy of the document with all sections, see nature.com/documents/nr-reporting-summary-flat.pdf

Life sciences study design

All studies must disclose on these points even when the disclosure is negative.

Sample size	Sample size was chosen based on experience of the investigators with similar experiments conducted multiple times and previously published.
Data exclusions	No data was excluded from the manuscript.
Replication	FACS analysis of protein binding to engineered HEK293 cell clones were performed multiple times (2-3) and with multiple clones per same gene engineering event (2-3). FACS data shown represents average of 2-3 independent experiments at a single concentration. All attempts at replication were successful.<br>SDS page analysis of purified mucin reporters were carried out 2-3 times with similar results.<br>For mucin cleavage assays different concentrations of the glycpeptidase StcE and mutant constructs were used and the SDS page gels were run 2x with similar results.<br>Immunohistochemistry was performed independently 2 times with fresh tissue samples with similar results.<br>ELISA assays were performed with antigen titrations (6-12), and the binding of select lectins and antibodies were repeated 2 times.
Randomization	Not applicable, but for cell binding assays in Fig. 3 KO clones with the same genetic engineering were randomly selected from available number of clones. No group allocation was performed, randomization is irrelevant to our study.
Blinding	No group allocation was performed, blinding is irrelevant to our study since there is no selection bias during the experiments or data collection.

Reporting for specific materials, systems and methods

We require information from authors about some types of materials, experimental systems and methods used in many studies. Here, indicate whether each material, system or method listed is relevant to your study. If you are not sure if a list item applies to your research, read the appropriate section before selecting a response.

Materials & experimental systems

n/a	Involved in the study	
☐	☒	Antibodies
☐	☒	Eukaryotic cell lines
☒	☐	Palaeontology and archaeology
☒	☐	Animals and other organisms
☒	☐	Human research participants
☒	☐	Clinical data
☒	☐	Dual use research of concern

Methods

n/a	Involved in the study	
☒	☐	ChIP-seq
☐	☒	Flow cytometry
☒	☐	MRI-based neuroimaging

Antibodies

Antibodies used	Commercial Primary Antibodies used:<br>APC- conjugated anit-FLAG (Biolegend) Cat: 637308 Clone: L5<br>mouse anti-6xHis antibody (R&D Systems) Cat:IC0501R-100UG<br>rabbit anti-GST (Invitrogen by Thermo Fisher) Cat: A-5800<br>Cy™3 AffiniPure Donkey Anti-Mouse IgG (ImmunoResearch) Cat: AB_2340813<br>Biotinylated PNA, VVA, MAL-II (Vector Lab) Cat: B-1075-5, B-1235, B-1265 respectively.<br>SiaFind™ Pan-Specific Lectenz®, Biotinylated Kit (Lectenz Bio) Cat: SK0501B<br>Goat anit- mouse IgG, Alexa Flour 647 (Invitrogen by Thermo Fisher) Cat: A21235<br>Goat anti-Rabbit IgG, Alexa Flour 647 (Invitrogen by Thermo Fisher) Cat: A21245<br>Goat anti-mouse IgG, Alexa Flour 488 (Invitrogen by Thermo Fisher) Cat: A10680

	Primary in house monoclonal Antibodies used:anti-Tn (5F4), anti-STn (TKH2), anti-Core1 (3C9), anti-MUC1 (5E10), anti-MUC1 (SM3), anti Tn-MUC1 (5E5), anti-Tn MUC2 (PMH1), anti-Tn MUC1/4 (6E3), anti-Tn MUC4 (3B11), anti-Tn MUC1 (2D9), anti-Core1 MUC1 (1B9), anti-MUC1 (5E10), anti-MUC1 (HMGF1).
Validation	Commercially purchased antibodies directed against standard protein tags were validated on cell lines transfected with proteins with or without the relevant tag.<br>APC- conjugated anit-FLAG (Biolegend). Statement on companies website" Each lot of this antibody is quality control tested by intracellular immunofluorescent staining with flow cytometric analysis".<br>mouse anti-6xHis antibody (R&D Systems) Cat:IC0501R-100UG is validated in Narimatsu et al., Mol Cell. 2019.<br>abbit anti-GST (Invitrogen by Thermo Fisher) is validated in Narimatsu et al., Mol Cell. 2019.<br>Biotinylated PNA, VVA, MAL-II (Vector Lab) is validated in Bull et al., PNAS. 2021<br>SiaFind™ Pan-Specific Lectenz® is validated in Bull et al., PNAS. 2021.<br><br>Monoclonal antibodies produced in-house were previously characterized by Steentoft et al., Glycobiology. 2019.<br>Used batches were validated in-house prior to using on cell lines expressing respective antigens.

Eukaryotic cell lines

Policy information about cell lines

Cell line source(s)	HEK293 6E cells (National Research Council, (Canada) were used.
Authentication	No specific authentication of cell lines used apart from separate handling of original obtained vials throughout entire project. Each individual engineered HEK293 clones were confirmed multiple times by HEK293 gene specific IDAA and Sanger sequencing in the target gene area(s).
Mycoplasma contamination	A representative set of growing cell lines in the lab selected randomly is subjected to mycoplasma screening bi-monthly, and within the last 10 yrs no infected cells have been found..
Commonly misidentified lines (See ICLAC register)	None of the cell lines used are listed in the ICLAC database.

Flow Cytometry

Plots

Confirm that:

- [x] The axis labels state the marker and fluorochrome used (e.g. CD4-FITC).
- [x] The axis scales are clearly visible. Include numbers along axes only for bottom left plot of group (a 'group' is an analysis of identical markers).
- [x] All plots are contour plots with outliers or pseudocolor plots.
- [x] A numerical value for number of cells or percentage (with statistics) is provided.

Methodology

Sample preparation	HEK293 cells were suspended in FACS buffer (PBS plus 1% BSA (w/v)) containing biotinylated lectin, mAbs or GST-tagged streptococcal adhesins for 1 h at 4°C in a 96 well plate, followed by washing 2x with FACS buffer and staining with appropriate secondary antibody for 30 minutes at 4°C. After subsequent 2x wash cells were resuspended in FACS buffer and fluorescence intensity was immediately measured.
Instrument	SONY SA3800 Spectral cell analyzer was used for all binding assays.
Software	FlowJo Version 10 was used.
Cell population abundance	Not applicable. Gating was performed only to exclude dead cells and doublets.
Gating strategy	The dead cells were excluded based on forward and side scatter area (FSC-A and SSC-A) parameter. Doublets were excluded based on FSC-H (height) and FSC-W (width) parameter.

- [x] Tick this box to confirm that a figure exemplifying the gating strategy is provided in the Supplementary Information.
